# Women’s enrollment in community-based health insurance and its determinants in Sidama national regional state, Ethiopia, 2024: A multilevel analysis

**DOI:** 10.1371/journal.pone.0316948

**Published:** 2025-02-03

**Authors:** Kare Chawicha Debessa, Keneni Gutema Negeri, Mesay Hailu Dangisso

**Affiliations:** 1 School of Public Health, College of Medicine and Health Sciences, Hawassa University, Hawassa, Ethiopia; 2 Ethiopian Public Health Institute, Addis Ababa, Ethiopia; University of Bologna, ITALY

## Abstract

**Introduction:**

Accessing affordable and high-quality healthcare remains a persistent challenge in low- and middle-income countries like Ethiopia. Community-based health insurance (CBHI) programs offer a promising solution to expand healthcare coverage and provide financial protection, particularly for vulnerable populations such as women. This study aimed to investigate the factors that influence CBHI enrollment among women in Sidama National Regional State, Ethiopia, using a multilevel analysis.

**Methods:**

A community-based cross-sectional study was conducted using a multistage sampling technique from December 15^th^ to January 12^th^, 2024, in the central Sidama zone, Sidama National Regional State. The study included 835 women aged 18 years and older, residing both in rural and urban areas. Face-to-face interviews were conducted using a pre-tested questionnaire to collect comprehensive data on socio-demographic, economic, and scheme-related factors. Data collection utilized the Open Data Kit mobile application, and data analysis was performed using Stata version 16, employing multilevel modified Poisson modeling to identify determinants of CBHI enrollment.

**Results:**

Among 845 samples, 835 women were interviewed, resulting in a response rate of 98.8%. Individual-level factors such as older age (APR = 1.02, 95%CI: 1.01–1.03, p<0.001), larger family size (APR = 1.09, 95%CI: 1.03–1.16, p = 0.001), and moderate wealth index (APR = 2.72, 95%CI: 1.28–5.79, p = 0.009) showed positive associations with CBHI enrollment, depicted a higher likelihood of individuals joining the insurance scheme. In addition, at the community level, higher rates of women’s literacy (APR = 1.73, 1.18–2.55, p = 0.005), and women’s autonomy (APR = 2.64, 95%CI: 1.50–4.65, p = 0.001) were positively correlated with CBHI enrollment.

**Conclusions:**

The study revealed that the enrollment rate among women in the CBHI scheme was 35%, indicating a need for improved outreach efforts. Significant factors that affected enrollment included older age, larger family size, and moderate wealth. Additionally, positive community-level influences such as higher literacy rates and increased autonomy for women contributed to higher enrollment. To improve CBHI enrollment, the target should focus on younger women and those from smaller families. Financial support, such as subsidies for low-income women, can also encourage participation. Investing in women’s literacy and empowerment programs will enable them to make informed health choices, thereby increasing enrollment. Finally, ongoing research is necessary to track enrollment trends and identify barriers. Utilizing qualitative methods will yield insights into women’s perceptions of CBHI, facilitating more effective strategies. Implementing these recommendations can enhance women’s access to healthcare through CBHI.

## Introduction

Universal health coverage (UHC) has emerged as a cornerstone aspiration within the global health discourse, recognized not only as a fundamental human right but also as a critical determinant for fostering social equity and improving health outcomes across diverse populations. The World Health Organization (WHO) defines UHC as the comprehensive provision of essential health services to all individuals without causing financial hardship [1].

This commitment to UHC has catalyzed a myriad of initiatives aimed at reducing the multifaceted barriers to healthcare access, particularly in low- and middle-income countries (LMICs), where systemic inequities often persist and manifest in pronounced disparities in health outcomes [2]. Within this framework, the exploration of innovative and sustainable financing mechanisms for health services becomes increasingly urgent, especially to address the enduring inequalities faced by vulnerable groups, including women and marginalized population groups.

Among the diverse strategies for health financing, community-based health insurance (CBHI) has garnered attention as a promising model aimed at increasing healthcare access and enhancing financial protection for marginalized communities. CBHI schemes, which are characterized by their community-oriented approach, encourage collective responsibility for health financing, enabling community members to pool resources and share the financial risks associated with healthcare utilization [3].

This model provides a structured alternative to the traditional reliance on out-of-pocket expenditures, which often leads to catastrophic health spending, particularly for the economically disadvantaged. CBHI programs hold particular significance in rural contexts where formal health insurance options are scarce, thus serving as a vital buffer against financial catastrophe for families seeking healthcare [4].

Ethiopia serves as an illustrative case for examining the dynamics of CBHI within the broader context of achieving UHC. The Ethiopian government has embarked on reforms aimed at expanding health coverage, recognizing the centrality of CBHI in its strategy to align with global health goals [5]. Since the introduction of CBHI programs in 2011, Ethiopia has experienced improvements in healthcare accessibility; however, significant barriers remain, particularly for women [6].

This is due to, in rural areas of Ethiopia, women often face a complex interplay of sociocultural, economic, and systemic challenges that impede their ability to participate in health insurance programs [7]. Such challenges necessitate an analysis of the factors influencing women’s enrollment in CBHI schemes, particularly within the Sidama National Regional State, a region distinguished by its socio-economic conditions and a predominantly rural population.

Recent empirical studies have illuminated the involvement of women in CBHI initiatives, revealing a paradox where women often serve as the primary caregivers and health decision-makers within families, yet encounter substantial barriers when attempting to access community-based health insurance. These barriers can be attributed to a variety of intersecting factors, including economic constraints, disparities in educational opportunities, and entrenched gender norms that limit women’s agency in health-related decision-making [8].

Despite the observed trend of increasing female engagement in CBHI enrollment, the existing body of literature is notably deficient in its attention to the region-specific dynamics that influence health insurance enrollment practices among women in the southern part of Ethiopia. Although numerous studies have analyzed the broader implementation of CBHI in other regions such as the northern part of Ethiopia, the unique socio-cultural and economic contexts of Sidama, southern Ethiopia, remain underexplored [9,10]. Without a thorough examination of these geographic areas, health interventions risk becoming misaligned with the specific issues & challenges faced by women in this region, thereby perpetuating existing inequalities in healthcare access.

Moreover, critical methodological gaps persist within the current body of research, particularly in how standard analytical models often simplify the complex interplay of multiple factors that influence women’s decisions regarding community-based health insurance enrollment. An understanding of individual & community-level dynamics requires the application of multilevel analytical tools, such as multilevel modeling techniques, which can more accurately illuminate the intricate relationships between individual-level characteristics (e.g., education, income) and community-level contexts (e.g., community-level women’s literacy, community-level women’s poverty) [11,12]. Addressing these methodological weaknesses is essential, as misinterpretations of these dynamics can lead to ineffective policy formulations that overlook the specific barriers encountered by women in their quest for health insurance.

Furthermore, the socio-cultural landscape in Ethiopia—where traditional gender roles often position men as the primary decision-makers—adds layers of complexity to the enrollment landscape for women. In various contexts, women experience diminished autonomy concerning financial and health-related decisions, a situation compounded by prevailing cultural norms that subordinate female agency [10]. This gendered perspective underscores the necessity for health policy frameworks to adopt a gender-sensitive lens, tackling both direct and indirect barriers to women’s enrollment in CBHI. Neglecting these gender dynamics not only perpetuates disparities in health access but also undermines broader efforts to achieve essential public health objectives [13].

The implications of these knowledge gaps warrant further attention. The failure to investigate the unique determinants influencing women’s enrollment in CBHI schemes hinders health authorities and policymakers from designing targeted interventions that engage women within health insurance initiatives. The lack of detailed data on the factors specific to women’s enrollment in Sidama, southern Ethiopia risks overlooking the perspectives and experiences of a critical segment of the population, thus perpetuating health inequities and stifling progress toward UHC. Addressing these complexities is not merely an academic pursuit; it represents a pressing imperative for public health policy aimed at achieving social equity and strengthening health system resilience.

Therefore, this study aimed to fill these knowledge gaps through an examination of the individual & community-level determinants influencing women’s enrollment in CBHI programs within Sidama National Regional State, southern Ethiopia. The anticipated findings aimed to provide valuable insights that can inform health policy and programming, ultimately supporting the design of gender-responsive strategies aimed at increasing women’s enrollment in community-based health insurance schemes. In pursuing this aim, the study hopes to contribute to collective efforts aimed at achieving universal health coverage in Ethiopia, with a particular focus on empowering women as key agents of health within their households and communities.

## Methods

### Study area

**Fig 1:**
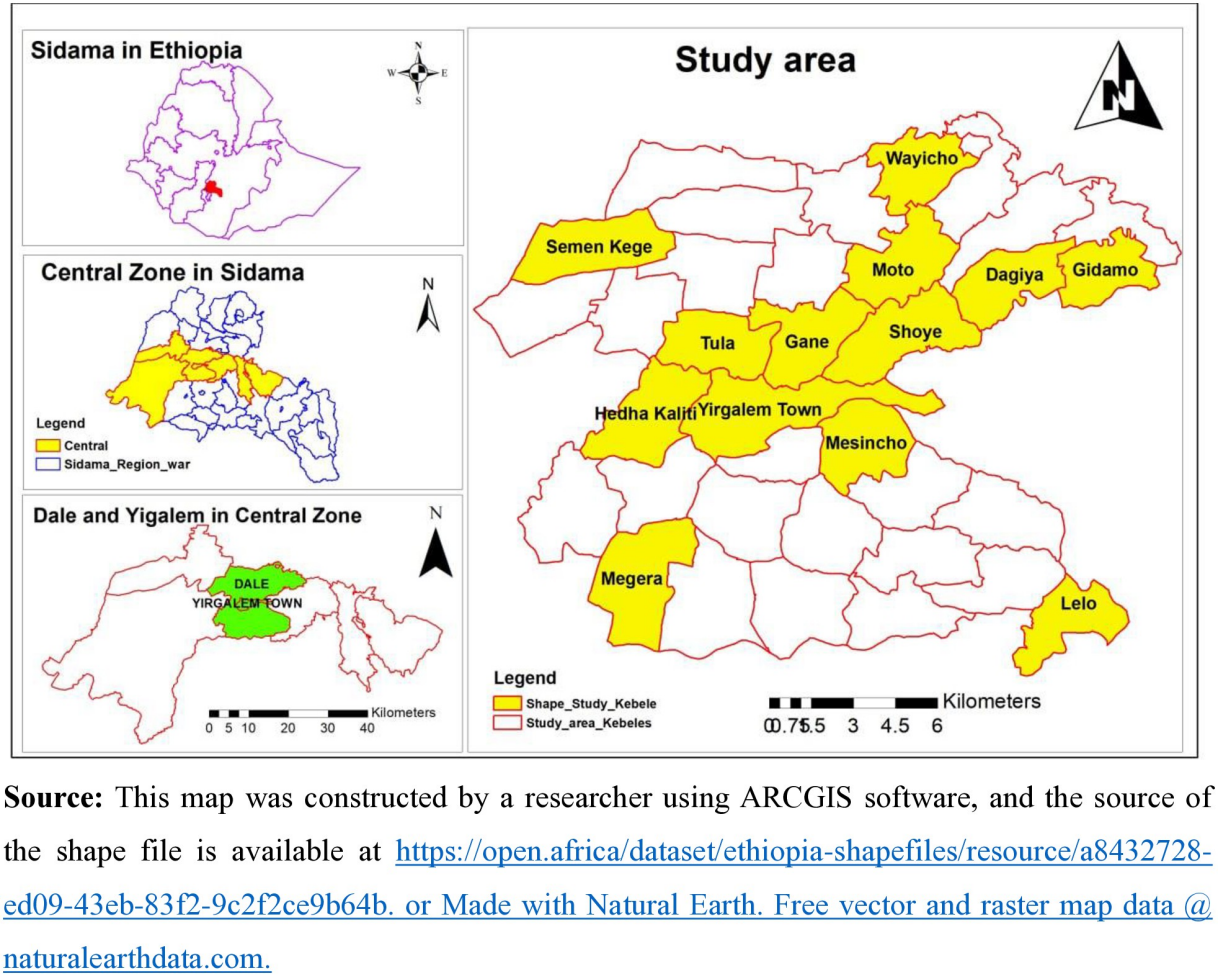
The administrative map of the Dale *woreda* and Yirgalem city administration in the central zone of Sidama National Regional State, Ethiopia.

The study was conducted in the central Sidama zone, Sidama region, Ethiopia. The Sidama region was newly established in June 2020 and is the second-smallest regional state in Ethiopia by land size but the sixth-largest in population size [14,15]. The Central Sidama Zone consists of six districts and one town administration, with a total population of 956,967 (2016 EFY) [16]. The study sites, Dale Woreda and Yirgalem City administration are located approximately 45 km south of the regional capital, Hawassa [17] (Fig 1).

This study purposefully selected Yirgalem City and Dale *Woreda* for several compelling reasons. Yirgalem City is home to the Yirgalem General Hospital, the first modern healthcare facility in the region, which has set a standard for healthcare delivery within the Sidama National Regional State [18]. Additionally, Yirgalem Hospital was among the initial sites for a healthcare financing initiative designed to generate revenue to improve services in southern Ethiopia [19]. This dual focus on CBHI and healthcare financing enabled an exploration of their interconnected effects. Historically, Yirgalem has been significant to the CBHI program, being one of the first 13 pilot sites launched in 2011, which allowed for an examination of progress and insights gained from its implementation [20].

Dale *woreda* was selected due to its proximity to Yirgalem and its diverse population, which presents various healthcare needs [21]. Assessing and understanding this diversity is crucial for analyzing factors influencing CBHI enrollment, particularly among women. Therefore, the selection of Yirgalem City and Dale *Woreda* enhanced the study and provided valuable insights for policymakers and healthcare providers intending to improve health insurance programs throughout the Sidama National Regional State. These findings significantly supported ongoing efforts to enhance healthcare access and equity in Ethiopia. However, households were randomly selected from these two sites to examine their CBHI enrollment status and identify key determinants.

### Study design and period

A community-based cross-sectional study design was carried out between December 15^th^ and January 12^th^, 2024. This study is based on the STROBE checklist and the checklist is provided as S1 File.

### Source and study population

The source population of this study was women residing in households of the Dale *Woreda* and Yirgalem city administration, which are situated in the central Sidama zone within the Sidama region. The study population consisted of women aged 18 years and older residing in households within the Dale *woreda* and Yirgalem city administrations, both located in the central Sidama zone of the Sidama region.

### Inclusion and exclusion criteria

Permanent residents of the study areas who had lived there for more than one year, and women aged 18 years and above, were included. Women who were unable to communicate due to illness or other reasons were excluded.

### Sampling and sample size

The sample size was calculated using OpenEpi software, based on a previous study’s proportion of 49% enrollment from Tigray region [22], with the assumption of a confidence limit of 5%, design effect 2, and a 10% non-response rate. Finally, we obtained a total sample of 845 households.

To achieve the necessary sample size for this study, a multi-stage sampling technique was employed, comprising the following steps. In the first stage, 14 *kebeles* were selected, including nine from Dale *woreda* (eight rural and one urban) and five from Yirgalem city administration (three rural and two urban). Simple random sampling was utilized to minimize bias; consequently, each *kebele* was marked on a map for easy identification during the data collection process. For the detailed definition of woreda administration, city administration, & *kebele*, please see the operational definitions section of this manuscript.

In the second stage, the estimated sample size was proportionately allocated to each *kebele* based on their respective population sizes. This approach ensured that the sample accurately reflected the demographics of the study areas, thereby enhancing the representativeness of the findings.

The third stage focused on selecting households within each chosen *kebele*. The number of sampled households was determined through proportional allocation relative to the population sizes, and simple random sampling was employed to ensure a representative sample.

**Fig 2:**
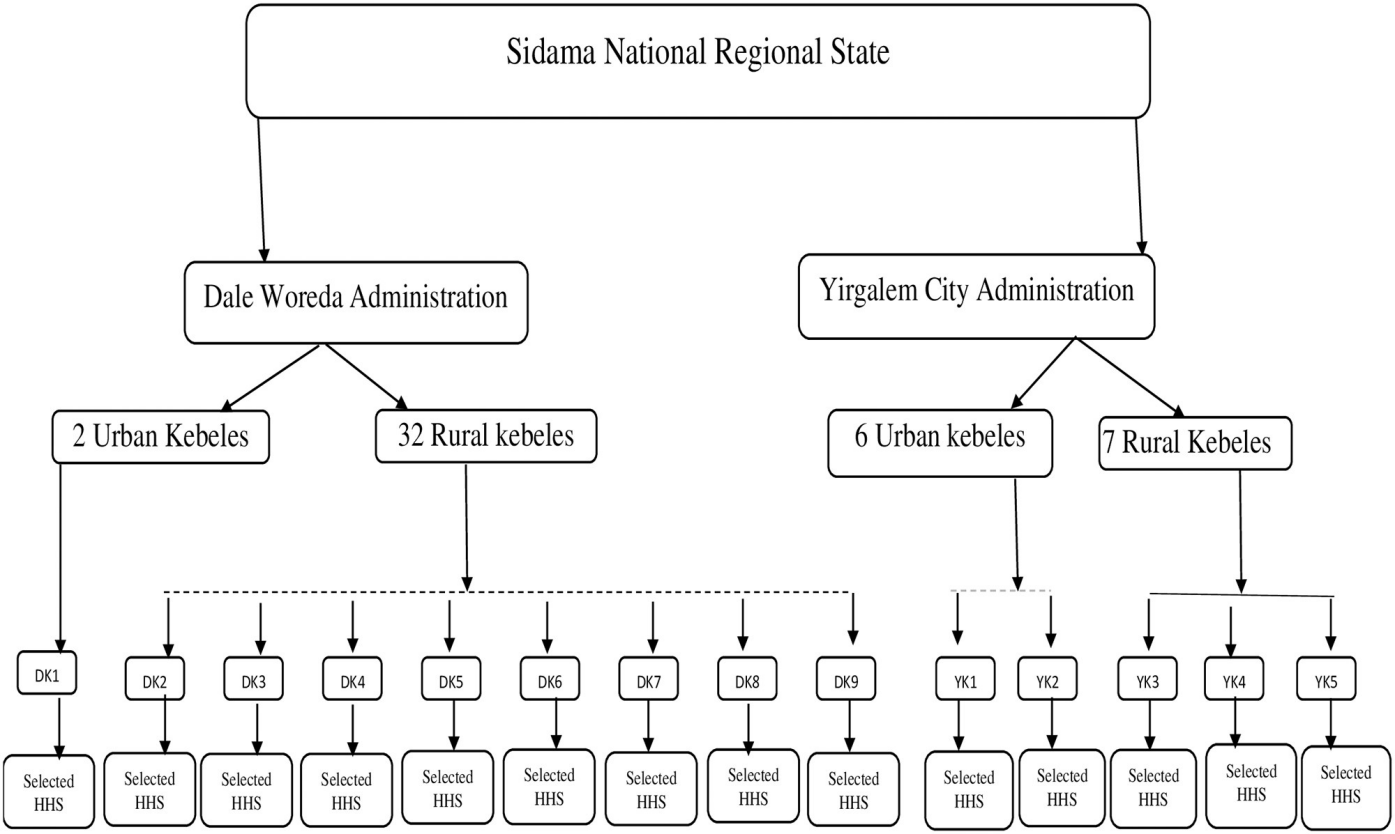
Schematic presentation of the sampling technique. Note: HHS means selected households for interview. On the other hand, DK 1, DK2, DK3, DK4, DK5, DK6, DK7, DK8, and DK9 represent the nine *kebeles* randomly selected from Dale *woreda*, while YKl, YK2, YK3 YK$, and YK5 represent the five *kebeles* randomly selected from Yirgalem city administration. Households were selected proportionally from each *kebele* based on their population size.

Finally, selected households were approached to identify eligible participants, primarily women due to their involvement in health-related issues. In cases where more than one eligible woman was present, the head of the household was consulted to determine which woman would participate. To maximize response rates, data collectors made up to three attempts to contact households in instances of absence, while a tracking sheet recorded essential information such as household ID, enumerator ID, date, and time of data collection (Fig 2).

Overall, this multi-stage sampling design not only facilitated a comprehensive approach to participant selection but also contributed to the reliability and validity of the data collected.

### Operational definition of terms

The term **age** denotes the chronological age of women as reported at the time of data collection.

**Family size** is defined as the total number of individuals residing in a household at the time of data collection.

**CBHI enrollment status** specifically indicates whether an individual was, at the time of data collection, a member of the community-based health insurance (CBHI) scheme. This status was recorded as a dichotomous variable, categorized as either "enrolled (yes)" or "not enrolled (no)," thereby clearly distinguishing between CBHI members and non-members.

**Women’s autonomy** refers to the proportion of women who were able to make decisions regarding participation in Community-Based Health Insurance (CBHI) and other livelihood matters, either independently or jointly with their husbands. Conversely, women who lacked this ability—whether independently or collaboratively—were classified as non-autonomous, when decision-making authority rested solely with male household members.

**Community-level women’s autonomy** serves as a proxy measure for assessing women’s decision-making power within the *kebel*e. Communities were categorized as autonomous or nonautonomous based on women’s involvement in decision-making related to livelihood issues. A community was considered to exhibit women’s autonomy if over 50% of women participated in decision-making processes-either independently or in conjunction with their husbands—at the *kebele* level. If this threshold is not met, indicating that fewer than 50% of women engaged in decision-making, the community is classified as lacking autonomy.

**Community-level women’s literacy** was determined by the proportion of women within a *kebele* who have achieved at least a primary level of education. If more than 50% of women have completed at least primary education, the community is classified as having a "high" proportion of literate women; otherwise, it is categorized as "low."

**Formal education** refers to structured educational programs that extend from primary through secondary and higher education, characterized by organized curricula implemented according to a defined calendar and timetable.

**Place of residence** was categorized into urban and rural classifications.

**Community-level poverty** was assessed by calculating the aggregated percentage of households within a *kebele* that belong to the poorest and poorer quintiles based on woman participant data. If this percentage exceeds 50%, the community is classified as having a "high" degree of poverty; otherwise, it is designated as "low."

**Recorded religion** refers to a classification system that categorizes women based on their religious affiliation, distinguishing between Protestants and other religious groups. This classification facilitated the analysis and understanding of religious demographics within the study population.

**Recoded ethnic group** denotes a classification system that categorizes women according to their ethnic affiliation, with those identifying as Sidama grouped in one category while other ethnic groups are consolidated into another category. This classification aided in analyzing ethnic demographics within the study population.

**Recoded education** refers to the categorization of women based on their educational background, specifically differentiating between those who have attended formal education and those who have not.

**Recoded marital status** categorizes women according to their marital status, including a category for married women and another for those with different marital statuses.

The term **employment** classifies women based on their employment status, encompassing categories for those currently employed and those who are not employed.

**Household head** categorizes households based on the gender of the household head as reported by women, distinguishing between male-headed and female-headed households.

The term **benefit packages** categorize essential healthcare offerings based on their relevance as reported by woman members of the Community-Based Health Insurance (CBHI). This categorization included both fully included benefit packages and those reported as partially included.

**CBHI premium contribution** refers to the classification of household contributions to the Community-Based Health Insurance (CBHI) program based on reports provided by women. These contributions were classified as either fair or not fair, reflecting women’s perceptions.

**Decisions** made by the Woreda Committee regarding the CBHI program can be classified into two categories: extremely transparent and somewhat transparent, based on women’s reports that reflect their perceptions of transparency in the decision-making process.

**CBHI decision inclusiveness** refers to whether decisions made regarding CBHI were classified as either extremely inclusive of relevant actors or partly inclusive of relevant actors, reflecting women’s perceptions regarding stakeholder involvement in CBHI decision-making processes.

The term **CBHI promotion adequacy** assesses the sufficiency of promotional efforts related to the Community-Based Health Insurance (CBHI) program, categorizing promotional activities reported by women as either adequate or not adequate.

The term **CBHI acceptable strategy** categorizes perceptions regarding the CBHI strategy program as either acceptable or not acceptable, based on perceptions made by women within their respective communities.

The **wealth index** represents an aggregate measure of household assets calculated using principal component analysis (PCA). It was assessed through inquiries about various asset components, including livestock, crop production, infrastructure (e.g., radio, modern bed, mattress, phone, water pump, modern stove), latrine facilities, housing conditions (e.g., number of rooms, roof type, floor type), and total farm size. The wealth index was computed using PCA techniques designed to reduce dimension in large datasets.

Consequently, our PCA excluded any assets or variables held by less than 5% or more than 95% of individuals in the sample. Ultimately, component factors or wealth index scores were ranked into five classes: lowest, second-lowest, middle, fourth-highest, and highest. Variables that did not meet assumptions-such as a Kaiser-Meyer-Olkin (KMO) measure below 0.5, commonalities below 0.5, or complex structures with high loading correlations (>0.4 on multiple components)—were also removed from PCA analysis.

**Woreda administration:** A woreda is a district-level administrative unit in Ethiopia that encompasses multiple *kebele*s, primarily rural, but occasionally a few urban. It acts as an intermediary between *kebeles* and regional administrations, responsible for planning and delivering public services such as education, healthcare, and infrastructure, governed by elected councils that influence local decision-making and resource allocation. In this case, Dale Woreda is considered a rural setting.

**Urban administration:** Urban administration refers to governance structures in Ethiopian cities and towns that integrate several *kebeles*, mainly urban with some or few rural. This administration handles urban services, economic development, and planning while addressing challenges like housing, transportation, and sanitation, thus playing a crucial role in local development and enhancing the quality of life for urban residents. In this case, Yirgalem is considered an urban setting.

***Kebele*:** A kebele is the smallest formal administrative unit in Ethiopia, akin to a neighborhood or village, organized under woreda or urban administrations. It represents the grassroots level of governance, overseeing local administration, community services, and the implementation of government policies. *Kebeles* typically comprise several thousand residents (approximately 500 to 1,000 households), serving as the primary link between the government and local populations.

In summary, *kebeles* form the grassroots governance level, woredas function as district units comprising multiple *kebeles*, and urban administrations govern urban areas, each integral to Ethiopia’s decentralized governance system.

### Study variables

The dependent variable in this study was the enrollment status in CBHI, which was measured using self-reported information from women and had a binary response (yes or no). The independent variables were classified into individual-level and community-level variables.

The individual-level variables included socioeconomic and demographic factors such as age, educational status, family size, household head, scheme-related factors, and household wealth index. The community-level variables included place of residence, literacy level of women in the community, poverty level in the community, and community autonomy.

### Data collection and quality assurance

A structured questionnaire, adapted from existing studies [23,24], served as the primary instrument for data collection (see S2 File). The survey was designed to enhance validity by including items from previously validated tools relevant to similar populations and constructs. The initial version of the questionnaire was drafted in English and subsequently translated into the local dialect, *Sidaamu Afoo*. This detail helps to underscore the credibility of our findings. The rationale for this dual translation process—first into the local dialect and then back to English—was to promote cultural relevance and ensure that participants could easily comprehend the content [25].

This approach aimed to minimize misunderstandings and enhance the overall integrity of the data collection process. To maintain the fidelity of meaning throughout the translation, we employed rigorous quality assurance measures.

Specifically, two bilingual experts facilitated the translation and back-translation process, ensuring that the original intent of the questions was preserved and culturally appropriate language was utilized. It is critical to note that all data were collected in the local dialect, *Sidaamu Afoo*. The data collectors were fluent speakers of this language, which enabled effective communication during the data collection process.

Before the comprehensive data collection, a pre-test of the translated questionnaire was conducted with a sample representing 5% of randomly selected women from *Hitata kebele*, *Tabor* sub-city, and Hawassa city. Feedback from both participants and data collectors was instrumental in refining the questionnaire, enabling us to enhance clarity, rectify any coding errors, and ensure cultural sensitivity. This iterative process ensured that the final version of the questionnaire was well-adapted for face-to-face interviews. To evaluate the reliability and validity of the data collection instruments, we conducted an assessment of internal consistency using Cronbach’s alpha, aiming for a threshold score exceeding 0.8 for multi-item scales. The final instrument achieved a reliability score of 0.9, indicating a high level of internal consistency.

Data were collected by twenty-five trained interviewers, each holding a bachelor’s degree in health sciences and fluent in *Sidaamu Afoo*. The interviews were conducted using the Open Data Kit (ODK) mobile application, with oversight provided by five experienced supervisors who held master’s degrees in public health. To ensure data quality, accuracy, completeness, and adherence to ethical standards, the study team implemented a comprehensive set of quality control measures. These included extensive training and pretesting of data collectors, conducting re-interviews, pre-testing, and daily monitoring to identify and rectify any issues related to labeling, incomplete responses, or formatting errors. Such meticulous efforts contributed to upholding the integrity and quality of this study.

### Data analysis

Before conducting the main analysis, we performed variable recoding, computations, and categorizations. The complete data set in which this manuscript is provided as S4 File. Consequently, for categorical variables, summary measures were expressed as absolute frequencies and percentages. In contrast, for numerical variables, the mean with standard deviation (SD) was used as a descriptive measure after checking the normality of the distribution [26].

The wealth index was calculated using principal component analysis (PCA) as a combined indicator of living standards. Accordingly, it was based on 42 questions related to ownership of selected household assets, such as house ownership, construction materials, number of rooms, agricultural land size, presence of livestock, cooking fuel types, and possession of improved sanitation and water facilities [27].

To estimate the prevalence ratios with 95% confidence intervals (CIs) for the determinants of the outcome of interest, a modified Poisson regression with robust standard error was employed. This technique is preferred over logistic regression when the prevalence of the outcome exceeds 20% [28–30]. Additionally, the prevalence ratio is easier to understand or communicate to non-epidemiologists than the odds ratio [31]. Furthermore, the odds ratio can overestimate the prevalence ratio, and the adjustment for confounding is not equivalent between the two measures [29].

Before conducting a multilevel analysis, the need for a multilevel model was examined by employing a random intercept model of mixed-effect multilevel logistic regression. This model also generates the intraclass correlation coefficient (ICC) to determine the necessity of a multilevel model. If the ICC value exceeds 5%, the multilevel analysis model becomes essential [32]. Additionally, in the multivariate analysis model, variables with p-values < 0.25 from bivariable analysis were included, along with other variables supported by literature, to account for potential confounding [33]. Moreover, effect modification was evaluated by sequentially introducing interaction terms. Similarly, multicollinearity among independent variables was evaluated using multiple linear regression with a variance inflation factor threshold of < 5 [34].

To account for the hierarchical nature of the data and reduce potential standard error underestimation using the ordinary regression model, a multilevel model that provided a robust standard error estimate was built [32]. Furthermore, to evaluate the necessity of a multilevel model, four models were assessed: Model 0 (empty model), Model 1 (with only individual-level variables), Model 2 (with only community-level variables), and Model 3 (with both individual- and community-level variables).

Finally, statistically significant associations were determined using adjusted prevalence ratios (APRs) with 95% CIs. An APR CI excluding 1 confirmed the association [35].

### Ethics statement

The institutional review board (IRB) at Hawassa University’s College of Medicine and Health Sciences granted this project ethical permission with reference number IRB/021/16. The Hawassa University School of Public Health, the Sidama Region Health Bureau, district health offices, and kebele administrators all provided a letter of support.

Before interviews, all study respondents who satisfied the inclusion criteria provided written informed consent. The study subjects were informed about the study’s goal, data gathering methods, voluntary participation, confidentiality, potential benefits, and risks before they signed informed written consent. Confidentiality was maintained throughout the whole data collection and storage process. Other than needing to spend forty minutes at the interview, there was no risk or hurt associated with taking part in this study. Certain personal details may cause a little discomfort. We did not want this to occur, though; therefore, each participant was given full authority to decline to answer any questions if it made them feel uncomfortable.

## Results

**Fig 3:**
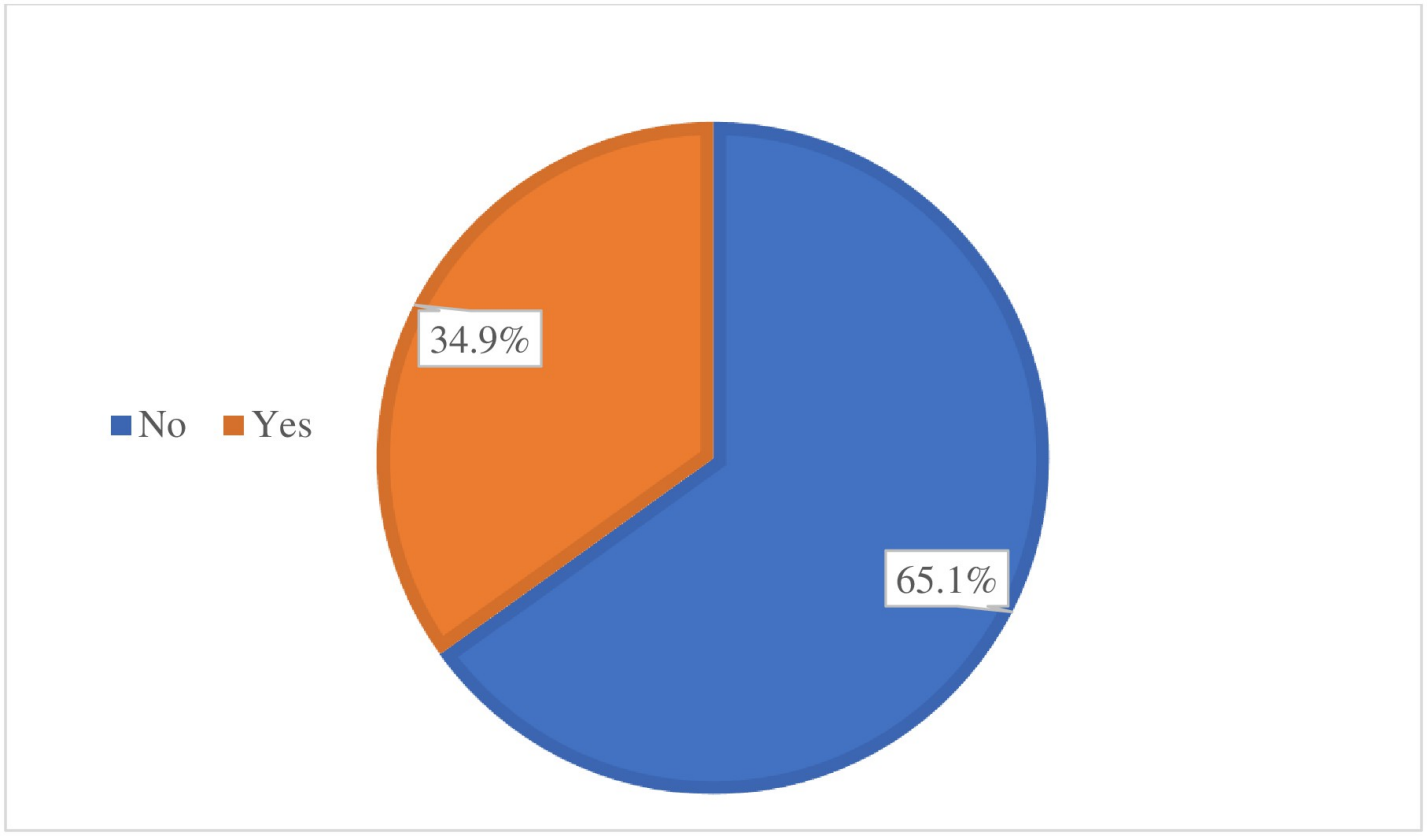
Community-based health insurance proportion among women in the central zone of Sidama region Ethiopia, 2024 (n = 835).

From a sample of 845 participants total of 835 women were included in the interviews, resulting in a response rate of 98.8%. Among the interviewed women, 291 had enrolled for CBHI, representing a proportion of 34.9% (34.9%, 95% CI: 31.69–38.15). In contrast, 544 (65.1%) women had not enrolled in CBHI (Fig 3).

The study participants were predominantly rural residents, with 484 (58%) living in rural areas and 351 (42%) residing in urban settings. Consequently, the respondents’ ages ranged from 18 to 80 years, with a mean of 38.72 (SD = ±14.105) years. Furthermore, the majority of participants, 737 (88.3%), identified as Protestant, and 797 (95.4%) belonged to the Sidama ethnic group.

Regarding household composition, 94 (11.3%) women interviewed were from female-headed households, while the remaining 741 (88.7%) were from male-headed households. Additionally, the majority of the women, 780 (93%), were married, and the mean family size was 4.82 (SD = ±1.468), with a range of 1 to 7 family members.

In terms of education, 468 (56%) of the study participants had received formal education, while 367 (44%) had not. Conversely, when examining the wealth index, 223 (26.7%) of the participants fell into the middle wealth category distribution, while the remaining 73.3% were distributed across the lowest 132 (15.8%), second 130 (15.6%), fourth 186 (22.3%), and highest 164 (19.6%) categories. Nonetheless, at the community level, the literacy rate among women was 394 (47.2%), the poverty level was 468 (56%) high, 367 (44%) low, and the women’s autonomy level was 468 (58.2%). For the details, see Table 1.

**Table 1:** Sociodemographic, economic, and scheme-related characteristics of the study participants in the central zone of Sidama region, Ethiopia, 2024 (N = 835).

Variables	Categories	N	%
**Residence**	Urban	351	42
	Rural	484	58
**Recorded religion**	Protestant	737	88.3
	Others	98	11.7
**Recoded ethnic group**	Sidama	797	95.5
	Others	38	4.6
**Wealth index**	Lowest	132	15.8
	Second	130	15.6
	Middle	223	26.7
	Fourth	186	22.3
	Highest	164	19.6
**Community-level women’s literacy**	Illiterate	441	52.8
	Literate	394	47.2
**Community-level women’s autonomy**	Not autonomous	404	48.4
	Autonomous	431	51.6
**Recoded education**	Not attended formal education	367	44
	Attended from education	468	56
**Women autonomy**	Un autonomous	373	44.7
	Autonomous	462	55.3
**Recoded_marital_status**	Married	780	93.3
	Others	55	6.6
**Employment**	Employed	502	60.1
	Not employed	333	39.9
**Community level poverty**	High	468	56
	Low	367	44
**Household head reported by women**	Male	741	88.7
	Female	94	11.3
**CBHI membership**	No	544	65.1
	Yes	291	34.9
**Benefit packages**	Relevant packages included	258	30.9
	Partly included	577	69.1
**CBHI premium fair**	Fair	385	46.1
	It is not fair	450	53.9
**CBHI decisions transparent**	Extremely transparent	270	32.3
	Somewhat transparent	565	67.7
**CBHI decisions inclusive**	Extremely inclusive	207	24.8
	Partly inclusive	628	75.2
**CBHI promotion adequate**	Adequate	420	50.3
	Not adequate	415	49.7
**CBHI acceptable strategy**	No	539	64.6
	Yes	304	36.4

*In recoded religion others means Orthodox, Catholic, Muslim, and Traditional.

*In recoded ethnic groups others means Amhara, Wolaita, Gurage, Siltie, and Oromo.

*In recoded marital status others means Never married, living together, Divorced or Separated, and Widowed.

### Random model information

The present study assessed the determinants of community-based health insurance (CBHI) membership among households nested within the *Kebele level*. To this end, the intraclass correlation (ICC) was calculated to quantify the degree of clustering of CBHI membership within the *Kebele-*level hierarchy.

The results in Table 2 indicated that the ICC at the *Kebele* level was estimated at 0.156, with a standard error of 0.052 and (95% CI: 0.078, 0.28). Consequently, approximately 15.6% of the total variance in CBHI membership was attributable to differences between the *Kebele* levels. For the details, see Table 2 below.

**Table 2:** The intraclass correlation coefficient.

Level	ICC	Std. Err	(95% confidence interval)
**Kebele**	0.156	0.052	0.078–0.28

This substantial ICC value indicates that households within the same *Kebele* were more similar in their CBHI membership status compared to households from different *Kebele*. As such, the independence assumption underlying standard regression models was violated, thereby warranting the use of a multilevel modeling approach to properly account for the hierarchical structure of the data [36]. The observed ICC of 0.156 (15.6%) revealed that a sizable proportion of the variability in CBHI membership is attributable to the *Kebele*-level clustering. Therefore, a multilevel modeling approach is warranted to accurately model the determinants of CBHI membership, given the hierarchical structure of the data [37].

### Community-based health insurance among women and associated factors in the central zone of Sidama region, Ethiopia, 2024 (N = 835)

The results of the multilevel modified Poisson regression analysis were presented as follows: Communities with higher levels of women’s autonomy exhibited a significantly greater likelihood of community-based health insurance (CBHI) membership compared to those with lower levels of autonomy, with an adjusted prevalence ratio of 2.6 (95% CI: 1.50–4.65, p = 0.001). This finding indicated that women in communities characterized by greater self-autonomy and decision-making power were 2.6 times more likely to enroll in CBHI than their counterparts in communities with less autonomy.

Moreover, communities with higher levels of women’s literacy demonstrated a significantly higher probability of CBHI membership relative to those with lower literacy levels among women (adjusted prevalence ratio = 1.7, 95% CI: 1.18–2.55, p = 0.005). This suggested that increased community-level women’s literacy was associated with a 74% greater likelihood of enrollment in CBHI compared to areas with lower literacy rates.

Additionally, when comparing women in the lowest wealth index category to those in the "middle wealth index" category, a significantly higher likelihood of CBHI membership was observed (adjusted prevalence ratio = 2.7, 95% CI: 1.28–5.79, p = 0.009). However, no significant differences were noted among the "second," "fourth," and "richest" categories. This finding implied that women classified within the middle wealth index category were approximately 2.7 times more likely to enroll in CBHI than those from the poorest wealth index households. While enrollment was anticipated among the poorest, barriers such as unaffordable premiums and limited awareness likely contributed to the enrollment gap between middle-income and the most disadvantaged groups.

Furthermore, older age was significantly associated with a higher probability of CBHI membership (adjusted IRR = 1.023 per year increase in age, 95% CI: 1.01–1.03, p < 0.001). Specifically, each additional year in a woman’s age was associated with a 2.3% increase in the likelihood of CBHI enrollment. Additionally, larger family size was significantly related to an increased likelihood of CBHI membership (adjusted prevalence ratio = 1.099 per unit increase in family size, 95% CI: 1.040–1.161, p = 0.001). This indicated that an increase of one member in family size was associated with a 9.9% higher probability of enrolling in CBHI (Table 3).

**Table 3:** Determinants of CBHI enrollment among women in the central zone of Sidama region, Ethiopia, 2024 (N = 835).

Variables		Are you a member of CBHI?		CPR (95% CI)	APR (95% CI)
		No	Yes		
**Age in years**				1.03(1.02–1.04)	1.02(1.01–1.03)
**Family size**				1.08(1.03–1.15)	1.1(1.04–1.16)
**Residence**	Urban	196(55.8)	155(44.2)	1	1
	Rural	348(71.9)	136(28.1)	0.87(0.65–1.17)	1.02(0.73–1.44)
**Wealth index**	Lowest	118(89.4)	14(10.6)	1	1
	Second	98(65.8)	51(34.2)	2.16(1.02–4.54)	1.81(0.90–3.57)
	Middle	84(38.7)	133(61.3)	2.66(1.24–5.70)	2.72(1.28–5.79)
	Fourth	126(72.4)	48(27.6)	1.67(0.71–3.93)	1.80(0.80–5.41)
	Highest	118(72.4)	45(27.6)	1.94(0.80–4.86)	2.14(0.92–5.00)
**Community-level women’s literacy**	Illiterate	338(71.3)	136(28.7)	1	1
	Literate	206(57.1)	155(42.9)	1.95(1.02–3.72)	1.73(1.18–2.55)
**Community-level women’s autonomy**	Not autonomous	354(81.6)	80(18.4))	1	1
	Autonomous	190(47.4)	211(52.6)	4.00(1.93–8.31)	2.64(1.50–4.65)
**Promotion adequate**	Adequate	199(47.4)	221(52.6)	1	1
	Not adequate	345(83.1)	70(16.9)	0.91(0.71_1.18)	0.91(0.70–1.22)
**Contribution fair**	Fair	171(44.4)	214(55.6)	1	1
	Not fair	373(82.9)	77(17.1)	0.94(0.67–1.33)	0.86(0.64–1.16)
**Decision transparent**	transparent	94(34.8)	176(65.2)	1	1
	Not transparent	450(79.6)	115(20.4)	0.76(0.60–0.97)	0.93(-0.72–1.20)
**Decision inclusive**	Include all times	66(31.9)	141(68.1)	1	1
	Include some times	478(76.1)	150(23.9)	0.80(0.64–1.01)	0.81(0.64–1.02)
**Packages adequacy**	Adequate	116(45)	142(55)	1	1
	Not adequate	428(74.2)	149(25.8)	0.92(0.75–1.11)	0.91(0.75–1.10)
**Acceptable strategy**	No	266(87.5)	38(12.5)	1	1
	Yes	278(52.4)	253(47.6)	1.93(1.02–3.64)	1.60(0.90–2.94)

*CPR means Crude Prevalence Ratio.

*APR means Adjusted Prevalence Ratio.

### Model selection

The details of the model selection description are provided in S4 File.

## Discussion

The primary aim of this study was to investigate the factors influencing the enrollment of women in community-based health insurance (CBHI) within the Central Sidama Zone, Ethiopia. The study findings revealed an enrollment rate of 34.9%. This result demonstrated a significant increase in comparison to the enrollment rates reported in prior studies conducted in Sidama of 12.8% [23], in overall Ethiopia of 20.2% [38], and in the 2019 Ethiopia Mini Demography and Health Survey of 33.13% [39]. Additionally, lower CBHI enrollment rates were observed among women in various sub-Saharan African countries: 8.5% [40], Nigeria (< 3% [41], Ethiopia 4.7% [42], and other East African countries (7.56% [43].

Moreover, the study specifically highlighted that the coverage of health insurance among married women in Mauritania was 8.7% [44]. A comprehensive Demographic and Health Survey conducted across 29 sub-Saharan African countries between 2010 and 2020 showed a coverage rate of 21.3% for health insurance among married women. It is important to note that Ghana exhibited the highest coverage rate of 66.7%, while Burkina Faso had the lowest coverage rate of 0.5%. Other countries’ coverage varied between these extremes [45]. In light of these variations, it is crucial to acknowledge that despite the differences, the enrollment rate in the Central Sidama Zone remained significantly higher compared to the rates reported in other countries. This highlighted the need for continued efforts to improve health insurance coverage and access in sub-Saharan Africa, specifically in Ethiopia.

Conversely, the findings of the study indicated that the enrollment rate in the Central Sidama Zone was lower in comparison to the enrollment rates reported in the North Shoa Zone 58.6% [46], the Ethiopia Demographic and Health Survey, which reported a CBHI enrollment rate of 43.2% among women [47], Western Gojam 58% [48], and Rwanda 77.2% [49]. Similarly, in Gabon, the coverage rate was 42.8% [40] and in Indonesia, the coverage rate was 40.0% [50]. These differences or low enrollment are partly explained by the web of challenges such as limited awareness and knowledge about the benefits of CBHI and low income and affordability of health insurance premiums [51].

On the other hand, age, family size, wealth index, community-level women’s autonomy, and literacy were significant determinants of CBHI enrollment among women in Central Sidama Zone, Ethiopia. Regarding age, the study found that older individuals had a higher probability of CBHI enrollment. Correspondingly, this finding is consistent with existing literature that suggests older age as a predictor of health insurance coverage [38,52–54]. This may be because older people are more afraid of impending illness than younger people. Evidence suggests that older women believe they are more susceptible to diseases as their age increases [38,55]. In addition, older individuals may have higher healthcare needs and perceive the value of insurance more strongly, leading to increased enrollment rates. Therefore, they get health insurance at a low cost to ensure safe health care utilization [55]. Conversely, a systematic review in low and middle-income countries revealed that younger individuals were willing to pay more than older individuals [56].

The study also revealed a positive relationship between family size and CBHI membership. Larger families were more likely to enroll in CBHI, indicating that family size played a role in enrollment decisions. Likewise, this finding aligns with previous research that has highlighted the influence of family size on health insurance enrollment [38,48,53,56]. Larger families may have a higher need for healthcare services and perceive insurance as a way to mitigate financial risks associated with healthcare expenses [57].

In addition, women with the middle wealth index category were more likely to enroll in community-based health insurance than the poorest wealth index household category [39,54,58,59]. While the "poorest" were expected to enroll, they likely faced barriers like unaffordable premiums and low awareness, contributing to the enrollment gap between the middle wealth index and the most disadvantaged groups [60]. However, this study is inconsistent with the study conducted in Ethiopia, where poor wealth status households were more likely to enroll in CBHI than rich households [61]. A study conducted in the western part of Ethiopia showed mixed results where households from the poor and middle wealth index were more likely to be enrolled in CBHI compared to those from the rich wealth index [62]. Another study conducted in Nepal showed the higher level of household economic status was associated with increased enrollment in health insurance programs with the second, third, fourth, and highest quintile of households [63].

The results indicated that communities with higher levels of women’s autonomy were more likely to participate in community-based health insurance (CBHI) enrollment. This finding aligned with existing literature that underscored the importance of women’s empowerment and decision-making in healthcare utilization [64]. It suggested that when women have greater autonomy and decision-making power, they are more inclined to enroll in CBHI programs [45].

However, this study is inconsistent with the study conducted in Rwanda. According to the Rwandan study, women with high decision-making power and those experiencing high economic empowerment were less likely to utilize health insurance compared to their less empowered counterparts [49]. This paradox could be attributed to several factors that differentiated the two contexts. One potential explanation was that empowered women, who were frequently well-educated and from wealthier backgrounds, might have faced fewer obstacles in accessing healthcare services [49].

As a result, they could have possessed the financial means to cover out-of-pocket healthcare costs, leading to a lesser dependency on health insurance. This situation depicted that increased empowerment did not necessarily correlate with a higher likelihood of seeking health insurance, as these women may have perceived themselves as capable of managing their health expenses without the need for insurance coverage [65].

These discrepancies between the study in Rwanda and the current findings underscored the complexity of women’s health insurance enrollment behavior. While higher levels of autonomy in some contexts could foster participation in CBHI programs, on the other hand, unique socio-economic landscapes and healthcare policies within different countries could significantly influence enrollment trends [66]. Thus, it became crucial to consider contextual factors, such as local healthcare infrastructure, cultural attitudes towards insurance, and the overall economic environment, when interpreting the relationship between women’s empowerment and health insurance enrollment [67].

Furthermore, the study found a positive association between community literacy and CBHI membership. Communities with higher levels of literacy or formal education attendance had a higher probability of enrollment in CBHI. Likewise, this aligned with previous literature that highlights the positive relationship between education and health insurance enrollment [68–71]. This could be partly because education enhances health literacy, improves understanding of insurance benefits, increases awareness of the importance of healthcare, and improves the income of individuals.

### Limitations of the study

While this study yields valuable insights, its limitations must be acknowledged. The cross-sectional design restricts the capability to establish causal relationships between outcome and predictor variables [72]. This necessitates caution in interpreting the data, as information collected at a single point may not accurately represent ongoing dynamics. As Wang and Cheng (2020) indicated, this design limits definitive conclusions about the factors influencing women’s enrollment in health insurance. Without longitudinal data, it is unclear whether the identified factors drive enrollment or if healthier individuals are more inclined to enroll, potentially resulting in an overestimation of positive influences due to confounding health outcomes [73].

Self-reporting introduces additional complexity, as participants may provide exaggerated or socially desirable responses [74]. Such biases can lead to inaccuracies, with individuals possibly overstating benefits like affordability while minimizing barriers such as lack of awareness or distrust, thus distorting the understanding of enrollment factors.

Moreover, sampling bias threatens the reliability of the findings [75]. If the sample does not adequately represent the diverse demographics of women in the study areas, particularly marginalized groups, conclusions may be misleading about the challenges faced by lower-income women. Additionally, reluctance to share sensitive socioeconomic information may skew participant responses, leading to overly optimistic perceptions [76].

Measurement error is another crucial concern [77]. Hence, the specific findings from Sidama may not extend to other nations due to unique cultural and socioeconomic factors. Lastly, if data collection coincided with health outreach campaigns, enrollment figures might be artificially inflated [78]. Thus, while the study offers insights into women’s enrollment in community-based health insurance, these limitations must be considered, as they affect the generalizability of the findings.

## Conclusion

This study identified a low enrollment rate of only 35% among women in the community-based health insurance (CBHI) program, highlighting an urgent need for enhanced outreach efforts to engage more women. The results from the multilevel modified Poisson regression analysis revealed significant associations between women’s autonomy, literacy levels, wealth index categories, age, family size, and CBHI membership.

Communities characterized by higher levels of women’s autonomy and literacy demonstrated a substantially increased likelihood of CBHI enrollment, suggesting that empowering women through improved decision-making and educational opportunities could promote greater participation in health insurance programs.

Moreover, the analysis indicated that women in the middle wealth index category were significantly more likely to enroll in CBHI compared to those in the lowest wealth category, underscoring the barriers faced by economically disadvantaged households. These barriers, including unaffordable premiums and limited awareness, contributed to the enrollment gap among different socioeconomic groups.

The positive correlation between older age and CBHI membership indicated that as women aged, their likelihood of enrolling in health insurance increased, potentially due to accumulated life experience and greater awareness of healthcare needs. Additionally, larger family size was associated with a higher probability of CBHI enrollment, suggesting that families prioritized health insurance as a means of safeguarding their collective well-being.

Overall, these findings emphasized the importance of addressing social determinants such as women’s autonomy and education to improve health insurance enrollment. By enhancing these factors, policymakers could expand access to healthcare services for vulnerable populations and further efforts toward achieving universal health coverage in Ethiopia.

## Supporting information

S1 File (DOCX)

S2 File (DOCX)

S3 File (SAV)

S4 File (DOCX)
