# Supplementary material for: Women’s enrollment in community-based health insurance and its determinants in Sidama national regional state, Ethiopia, 2024: A multilevel analysis
Source: PLoS One. 2025 Feb 3;20(2):e0316948. doi: 10.1371/journal.pone.0316948 (PMC11790101; doi:10.1371/journal.pone.0316948)
Supplement: S1 File — (DOCX) [file pone.0316948.s001.docx]

**Questionnaire**

**Participant information sheet**

Good morning/afternoon?

My name is---------------. I am from Hawassa University and researching community-based health insurance among women in Sidama Regional State.

1. Objective: to determine the CBHI status and its determinants in the Sidama national regional state, Ethiopia.

You are selected randomly as a potential participant during this study as a subject.

Potential risks: There will be no potential risk that may cause any harm to study participants.

Benefits: There are no financial benefits associated to this study. However, by taking part during this study, you may contribute to improve the performance of the community-based health insurance among women groups.

Confidentiality: All information given by you will be kept strictly confidential. Your participation shall be voluntary and not obligated to answer any question which you do not wish to answer. If you feel any discomfort to the questionnaire, please feel free to drop at any time. This questionnaire will take approximately 25 minutes.

Are you willing to participate in the study?

1- Yes 2 - No

If the answer is yes, thanks! Continue with the interview.

If the answer is no, Thanks! Never force or reinforce study participants in the study

**Written consent form participants**

Hawassa University

I have understood all about the objective and the process of the study. My participation is voluntary and not obligated to answer any question which I do not know or do not wish to answer. I also understood that all information given by me will be kept strictly confidential. Therefore, I am willing to participate in this study.

Study participant sign _____________________________ date _____________

Data collector name: _________________________________________Signature _________

Date of interview ----------- Month --------------- /2015 E. C.

Supervisor’s Name ___________________________________________ Signature________

Checked date________________ /2015 E.C.

Contact Address of the Principal investigator

Name: Kare Chawicha

E-Mail: [kare.debessa@gmail.com](mailto:abigiatenaw@gmail.com)

Cell-Phone: +251900505922

Annex 4: English Version questionnaires

Hawassa University, School of Public Health

This questionnaire is designed to census community-based health insurance among women from selected woredas in Sidama Regional State, Ethiopia.

Woreda: ______________ Kebele code___________ Household code ______________

Section I: Background characteristics of the study participants

| S. No | Question | Response |
| --- | --- | --- |
| 1.1 | How old are you? | ____years (in completed year) |
| 1.2 | What is your religion? | 1. Orthodox  2. Catholic  3. Protestant  4. Muslim  5. Traditional  6. Other (Specify)___________ |
| 1.3 | What is your ethnic group | 1. Sidama  2. Amhara  3. Wolaita  4. Gurage  5. Siltie  6. Others (specify)____________ |
| 1.4 | What is your marital status | 1. Never married  2. Married  3. Living together  4. Divorced/separated  5. Widowed |
| 1.5 | Residence | 1. Urban  2. Rural |
| 1.6 | Kebele | Mention: __________________ |
| 1.7 | With whom you are living now?  (Circle all apply) | 1. Husband  2. Family member  3. Alone  4. Relatives  5. Friends or peers  6. Others (specify)___________ |
| 1.8 | Who makes final decisions at household level? | 1.husband  2.wife  3. Both |
| 1.9 | Who is household head | 1. male  2. female |
| 1.10 | What is the number of your family size? | 1. One  2. Two  3.Three  4. Four  5. Five  6. Six  7. Seven and above |
| 1.11 | What is your educational level? | 1. Unable to read and write  2. Primary education  3. Secondary education  4. Diploma and above |
| 1.12 | What is your employment status? | 1.Employed  2.Not employed |
| 1.13 | What is your occupation? | 1. Government employee  2. Private  3. Domestic worker  4. Self employed  5. Unskilled manual  6. Unpaid family member  7. non-governmental organization  8. Other (specify)____________ |

Section II: Community based health insurance related questions

| S. No | Question | Response |
| --- | --- | --- |
| 2.1 | Currently are you a member of the CBHI? | 1. Yes, if yes skip to question 2.5  2. No |
| 2.2 | If you are not a member, what is (are) the reason(s)? Select all that apply | 1. Lack of knowledge 2. Lack of money 3. Because of bad image 4. Some inconveniences 5. Lack of trust 6. Because I afford health cost without difficulties |
| 2.3 | Would you like to join the CBHI scheme in the future? | 1. Yes 2. No 3. Not decided yet |
| 2.4 | If yes, how certain to join the scheme? | 1. Certainly join 2. I do not know when to join |
| 2.5 | If you are member, for how long have you been member of the CBHI? | 1. up to one year  2. up to two years  3. up to three years  4. up to four years  5. up to five years  6.up to six and more years |
| 2.6 | Are you contributing premium to the scheme regularly? | 1.Regularly  2.With some interruptions  3. Ceased contribution |
| 2.7 | Have you ever dropped out of CBHI? | 1.Yes  2.No |
| 2.8 | If yes, what were the reasons for the drop outs? More than one answer may apply | 1.Affordability (lack of money)  2.Lack of medications and drugs at health facility  3.Lack of laboratory services  4.Bad perception about the scheme  5.Poor behavior of the staffs  6.Poor service quality  7.Lack of transparency at the scheme  8.Lack of inclusiveness in decision making  9. Because I got alternate health financing opportunity |
| 2.9 | when did you drop out, please specify the year in Ethiopia calendar | 1.2014  2.2013  3.2012  4.2011  5.2010 and before |
| 2.10 | Have you ever been selected as a committee member to the CBHI? | 1.Yes  2.No |
| 2.11 | Who is the final decision maker of the scheme at woreda level? | 1.Woreda administration only  2.The woreda scheme committee  3.The general assembly of the scheme  4.The regional health bureau |
| 2.12 | Have you or any one of the family members ever sick during last 12 months? | 1.Yes  2. No |
| 2.13 | Have you or any one of your family members visited health facility during last 12 months? | 1.Yes  2.No |
| 2.14 | If yes, how frequently have you and/ or your family have visited? | ----------------times |
| 2.15 | Which institution have you visited? | 1. Government health facility  2. Private health facility  3. None governmental health facility  4. Traditional healers |
| 2.16 | If you or your family members treated in any one of the above facilities, what was the treatment outcome? | 1.Cured  2. Still sick  3. disability  4. On follow up  5. death |
| 2.17 | Approximately how far is the nearest health facility on walking from your home? | 1. Half an hour  2. half to an hour  3. more than one hour |
| 2.18 | On average, how long you or your family members waited at health facility (in hours)? | 1. Less than 30 minutes  2. 30 minutes to an hour  3. One to two hours  4. Two to three hours  5. Three to three hours  6. More than four hours |
| 2.19 | Accessible and convenience to reach out health facility | 1. Strongly dissatisfied 2. Dissatisfied 3. Neutral 4. Satisfied 5. Strongly satisfied |
| 2.20 | The reception at the card room | 1. Strongly dissatisfied 2. Dissatisfied 3. Neutral 4. Satisfied 5. Strongly satisfied |
| 2.21 | The care provided as outpatient services | 1. Strongly dissatisfied 2. Dissatisfied 3. Neutral 4. Satisfied 5. Strongly satisfied |
| 2.22 | The care provided as inpatient services | 1. Strongly dissatisfied 2. Dissatisfied 3. Neutral 4. Satisfied 5. Strongly satisfied |
| 2.23 | With the laboratory services | 1. Strongly dissatisfied 2. Dissatisfied 3. Neutral 4. Satisfied 5. Strongly satisfied |
| 2.24 | Explanation about diagnosis and treatment options | 1. Strongly dissatisfied 2. Dissatisfied 3. Neutral 4. Satisfied 5. Strongly satisfied |
| 2.25 | Treatment with respect and dignity | 1. Strongly dissatisfied 2. Dissatisfied 3. Neutral 4. Satisfied 5. Strongly satisfied |
| 2.26 | Health facility cleanliness and neatness | 1. Strongly dissatisfied 2. Dissatisfied 3. Neutral 4. Satisfied 5. Strongly satisfied |
| 2.27 | Privacy and confidentiality maintained | 1. Strongly dissatisfied 2. Dissatisfied 3. Neutral 4. Satisfied 5. Strongly satisfied |
| 2.28 | Information provided on medication and its side effects | 1. Strongly dissatisfied 2. Dissatisfied 3. Neutral 4. Satisfied 5. Strongly satisfied |
| 2.29 | With scheduling and waiting times | 1. Strongly dissatisfied 2. Dissatisfied 3. Neutral 4. Satisfied 5. Strongly satisfied |
| 2.30 | Participation in treatment decisions | 1. Strongly dissatisfied 2. Dissatisfied 3. Neutral 4. Satisfied 5. Strongly satisfied |
| 2.31 | Management of pain effectively | 1. Strongly dissatisfied 2. Dissatisfied 3. Neutral 4. Satisfied 5. Strongly satisfied |
| 2.32 | The overall attitude of healthcare providers | 1. Strongly dissatisfied 2. Dissatisfied 3. Neutral 4. Satisfied 5. Strongly satisfied |
| 2.33 | The overall quality of healthcare received | 1. Strongly dissatisfied 2. Dissatisfied 3. Neutral 4. Satisfied 5. Strongly satisfied |
| 2.34 | Recommend this health facility to others | 1. Completely disagree 2. Disagree 3. Neutral 4. Agree   Completely agree |
| 2.35 | What experience do you have with ambulance services? | 1. Readily available at any time  2. Respond some time  3. Ambulance service is not available at all |
| 2.36 | Is adequate water source available in the health facility? | 1. Yes  2. No  3. I don’t know |
| 2.37 | Is on grid power connected to the health facility? | 1.Yes  2.No  3.I don’t know |
| 2.38 | Which critical services you lacked from the health facility? (More than one answer may apply) | 1. Skilled health care provider 2. Outpatient services 3. In patient services 4. Surgical services 5. Medication and drugs 6. Laboratory services 7. All of the above are not adequate |
| 2.39 | Which kind of services were you or your family offered in the health facility? (More than one answer may apply) | 1. Outpatient services  2. Inpatient Services  3.Referral services |
| 2.40 | Have the CBHI improved the quality of health care? | 1.Extremely improved  2.Partly improved  3.partly deteriorated  4.Extremely deteriorated |
| 2.41 | How certain you continue with membership of CBHI | 1.Certainly continue  2. Certain based on some condition’s fulfilment  3. Quite immediately  4. I don’t know  5. I will decide in consultation with my husband |
| 2.42 | Do you think that benefit packages of the health care services are adequately covered by the CBHI? | 1.Agree that relevant benefit packages are adequately included  2.Relevant packages are not adequately included |
| 2.43 | Do you think the premium contribution to the CBHI is fair? | 1.Is fair  2.It is not fair |
| 2.44 | Do you think that the CBHI decision making process is transparent? | 1.Transparent  2. Not transparent |
| 2.45 | Do you think that the CBHI decision making process inclusive all relevant actors? | 1.Inclusive at all times  2.It includes some times |
| 2.46 | Is advocacy and promotion conducted by the scheme adequate? | 1.It is adequate  2.It is not adequate |
| 2.47 | Do you believe that access to CBHI will improve your access to healthcare services in your community? | 1. Yes  2. No |
| 2.48 | Do you believe that being enrolled in the CBHI plan will make health care more affordable for you and the members of your household? | 1. Yes  2. No |
| 2.49 | From either your experience or understanding, how would you rate the potential financial protection by community-based health insurance against the cost of illness? | 1. None  2. Low  3. Medium  4. High |
| 2.50 | What do you think is the potential level of access by households to affordable healthcare due to community-based health insurance? | 1. None 2. Low 3. Medium 4. High |
| 2.51 | How would you rate the potential of community-based health insurance to improve household health consumption patterns by ensuring that healthcare costs are reduced? | 1. None 2. Low 3. Medium 4. High |
| 2.52 | How would you rate the potential of CBHI to improve the quality of services provided by healthcare givers? | 1. None 2. Low 3. Medium 4. High |
| 2.53 | How would rate the potential of CBHI to ensure constant availability of drugs at health facilities in your community? | 1. None 2. Low 3. Medium 4. High |
| 2.54 | Is community-based health insurance acceptable to you as a strategy for paying for health care in this area? | 1. Yes 2. No |
| 2.55 | Would you advocate about the CBHI to others? | 1. Yes, I do 2. No, I don’t |

Section III: Wealth index attributes

| S. No | Question | Response | | |
| --- | --- | --- | --- | --- |
| 3.1. | What is the main source of drinking water for members of your household? | Piped water:   1. Piped into dwelling 2. Piped to yard/plot 3. Public tab/stand pipe 4. Borehole 5. Dug well: 6. Protected well 7. Unprotected well 8. Protected spring 9. Unprotected spring   Surface water:  River/pond/stream | | |
| 3.2. | What is the main source of water used by your household for other purposes such as cooking and hand washing? | 1. Piped water: 2. Piped into dwelling 3. Piped to yard/plot 4. Public tab/standpipe 5. Borehole 6. Dung well: 7. Protected well 8. Unprotected well 9. Water from spring:   Protected spring  Unprotected spring  Surface water:  River/pond/stream | | |
| 3.3 | Where is that water source located? | In own dwelling  In own yard/plot  Elsewhere | | |
| 3.4 | How long does it take to go there, get water, and come back? | Minutes __________  Don’t know | | |
| 3.5 | In the past two weeks, was the water from this source not available for at least one full day? | Yes  No  Don’t know | | |
| 3.6 | Do you do anything to the water to make it safer to drink? | Yes  No | | |
| 3.7. | What do you usually do to make the water safer to drink? Record all mentioned | Boil  Add bleach/chlorine  Strain through a cloth  Use water filter (Sand/composite/etc.)  Solar disinfection  Let it stand and settle | | |
| 3.8. | What kind of toilet facility do members of your household usually use? | 1. Flush or pour-flush toilet 2. Flush to a piped sewer system 3. Flush to septic tank 4. Flush to a pit latrine 5. Flush to somewhere else 6. Flush, don't know where 7. Pit latrine 8. Ventilated improved pit latrine 9. Pit latrine with slab   Pit latrine without slab/open pit  composting toilet  bucket toilet  Hanging toilet/hanging latrine  No facility/bush/field | | |
| 3.9 | Do you share this toilet facility with other households? | Yes  No | | |
| 3.10 | Including your household, how many households use this toilet facility? | Less than 10  10 or more  Don't know | | |
| 3.11 | Where is this toilet facility located? | In own dwelling  In own yard/plot  Elsewhere | | |
| 3.12 | What type of fuel does your household mainly use for cooking? | 1. Electricity 2. Liquid petroleum gas 3. Natural gas 4. Biogas~~~͠ 5. Kerosene 6. Charcoal 7. Wood 8. Straw/grass 9. Agricultural crop   Animal dung  No food cooked in house | | |
| 3.13 | Is the cooking usually done in the house, in a separate building, or outdoors? | In the house  In separate building  Outdoors  Other specify______________ | | |
| 3.14 | Do you have a separate room which is used as a kitchen? | Yes  No | | |
| 3.15 | Who is the owner of the house? | Me  Rental  Family  Relative | | |
| 3.16 | How many rooms in this household are used for sleeping? | There are --------Rooms | | |
| 3.17 | The main material of the roof of the house? | Natural roofing (no roof, mud, and sod)  Rudimentary roofing (rustic mat/plastic sheet, reed/bamboo, wood planks, and cardboard)  Finished roofing (metal/corrugated iron, wood, calamine/cement, ceramic tiles, roofing shingles) | | |
| 3.18 | The main material of the floor of the house? | Natural floor (Earth/sand, dung)  Rudimentary floor (wood planks, and palm/bamboo)  Finished floor (parquet or polished wood, vinyl or asphalt strips/ plastic tiles, cement, ceramic tiles, carpet) | | |
| 3.19 | Does this household own any livestock, herds, other farm animals, or poultry? | Yes  No | | |
| 3.20 | How many of the following animals do this household own?  If none, record '00'.  If 95 or more, record '95'.  If unknown, record '98'. | Cows bulls_______________  Other cattle______________  Horses____________  Donkeys_______  Mules ____  Camels________  Goats _________  Sheep _________  Chickens/poultry ______  Beehives ___________ | | |
| 3.21 | Do you have separate rooms for cattle? | Yes  No | | |
| 3.22 | Does any member of this household own any agricultural land? | Yes  No | | |
| 3.23 | How many hectares of agricultural land do members of this household own? | ___________hectares | | |
| 3.24 | Does any member of the have any one of these? |  | Yes (1) | No (0) |
|  |  | Electricity  Radio  Television  Non-mobile telephone  Computer  Refrigerator  Table  Chair  Bed with spring matters  Electric mitad  Kerosene  lamp/pressure Lamp-- |  |  |
| 3.25 | Does any member of this household own? |  | Yes (1) | No (0) |
|  |  | Watch  Mobile phone  Bicycle  Motorcycle/scooter  Animal-drawn cart  Car/truck  Boat with motor  Baggage |  |  |
| 3.26 | Does any member of this household have a bank account? | Yes  No | | |
| 3.27 | Does any member of this household have microfinance account? | Yes  No | | |
| 3.28 | How often does anyone smoke inside your house?  Would you say daily, weekly, monthly, less often than once a month, or never? | Daily  Weekly  Monthly  Less often than once a month  Never | | |

Thank you very much!

I would like to express my gratitude to the authors for their efforts in preparing this manuscript and to the editorial team for the opportunity to review this valuable manuscript. I look forward to potential improvements in the future. Thank you.

Asabu, M. D. & D. K. Altaseb (2021) The trends of women's autonomy in health care decision making and associated factors in Ethiopia: evidence from 2005, 2011 and 2016 DHS data. *BMC Womens Health,* 21**,** 371.

Bayeh, E. (2016) The role of empowering women and achieving gender equality to the sustainable development of Ethiopia. *Pacific Science Review B: Humanities and Social Sciences,* 2**,** 37-42.

Budu, E., A.-A. Seidu, E. K. Armah-Ansah, F. Sambah, L. Baatiema & B. O. Ahinkorah (2020) Women’s autonomy in healthcare decision-making and healthcare seeking behaviour for childhood illness in Ghana: Analysis of data from the 2014 Ghana Demographic and Health Survey. *PloS one,* 15**,** e0241488.

Coles, E., M. Wells, M. Maxwell, F. M. Harris, J. Anderson, N. M. Gray, G. Milner & S. MacGillivray (2017) The influence of contextual factors on healthcare quality improvement initiatives: what works, for whom and in what setting? Protocol for a realist review. *Syst Rev,* 6**,** 168.

Cornish, H., H. Walls, R. Ndirangu, N. Ogbureke, O. M. Bah, J. F. Tom-Kargbo, M. Dimoh & M. Ranganathan (2021) Women’s economic empowerment and health related decision-making in rural Sierra Leone. *Culture, health & sexuality,* 23**,** 19-36.

Dahab, R. & D. Sakellariou (2020) Barriers to accessing maternal care in low income countries in Africa: a systematic review. *International journal of environmental research and public health,* 17**,** 4292.

Dhufera, H. T. 2024. Identifying Indigent Households for Subsidy and Exemption of Community Health Insurance Premium in Ethiopia: Considerations of Technical Approaches and Implementation Challenges.

Dworkin, S., M. Gandhi & P. Passano. 2017. *Women's empowerment and global health: A twenty-first-century agenda*. Univ of California Press.

Fuller, R. & J. Lain (2020) Are female-headed households less resilient? Evidence from Oxfam’s impact evaluations. *Climate and Development,* 12**,** 420-435.

Htun, N. M. M., Z. L. Hnin & W. Khaing (2021) Empowerment and health care access barriers among currently married women in Myanmar. *BMC Public Health,* 21**,** 139.

Idris, I. B., A. A. Hamis, A. B. M. Bukhori, D. C. C. Hoong, H. Yusop, M. A.-A. Shaharuddin, N. A. F. A. Fauzi & T. Kandayah (2023a) Women’s autonomy in healthcare decision making: a systematic review. *BMC Women's Health,* 23**,** 643.

Idris, I. B., A. A. Hamis, A. B. M. Bukhori, D. C. C. Hoong, H. Yusop, M. A. Shaharuddin, N. Fauzi & T. Kandayah (2023b) Women's autonomy in healthcare decision making: a systematic review. *BMC Womens Health,* 23**,** 643.

Moonzwe Davis, L., S. L. Schensul, J. J. Schensul, R. K. Verma, B. K. Nastasi & R. Singh (2014) Women's empowerment and its differential impact on health in low-income communities in Mumbai, India. *Glob Public Health,* 9**,** 481-94.
