# Supplementary material for: Women’s enrollment in community-based health insurance and its determinants in Sidama national regional state, Ethiopia, 2024: A multilevel analysis
Source: PLoS One. 2025 Feb 3;20(2):e0316948. doi: 10.1371/journal.pone.0316948 (PMC11790101; doi:10.1371/journal.pone.0316948)
Supplement: S4 File — (DOCX) [file pone.0316948.s004.docx]

**Selection of Model Fitness**

Consequently, the model that best fit the data was selected by considering Akaike's information criteria (AIC), Bayesian information criteria (BIC), and log-likelihood with a likelihood ratio test. Choosing the model with the lowest values for these criteria and a statistically significant likelihood ratio test can indicate the best-fitting model [1]. The results of the model comparison and selection process are presented in the Table 4 below. Based on the statistical evidence, the "Individuals and community variables" model was identified as the best fit model.

**Log-likelihood (LL)**

The "individuals and community variables" model had the highest log-likelihood value of -453.90, indicating a better goodness of fit to the data compared to the other models [2].

**Akaike Information Criterion (AIC)**

The "individuals and community variables" model had the lowest AIC value of 933.80, which was lower than all the other models. Lower AIC values indicate better model fit, considering both the goodness of fit and the complexity of the model. The AIC statistic penalizes model complexity, favoring the most parsimonious model that adequately explains the data [3].

**Bayesian Information Criterion (BIC)**

The "individuals and community variables" model also had the lowest BIC value of 995.25, which was lower than all the other models. Lower BIC values likewise indicate better model fit, with a stronger penalty for model complexity compared to AIC. The BIC statistic imposes a heavier penalty for additional parameters, making it more conservative in selecting the optimal model [4].

The "individuals and community variables" model included both individual-level and community-level predictors, providing a more comprehensive understanding of the factors associated with the outcome. Furthermore, the model complexity, as indicated by the degrees of freedom, was reasonable and not overly complex, suggesting the model was not overfit to the data [5].

Based on the statistical evaluation of the log-likelihood, AIC, and BIC values, the "individuals and community variables" model was identified as the best fit model. This model brought a favorable balance between explanatory power and model complexity, making it the most suitable choice for further analysis and interpretation (Table 4).

**Table 4: Model comparison and** s**election criteria**

| Types of the models | Model selection criteria and the results | | |
| --- | --- | --- | --- |
|  | LL (model) | AIC | BIC |
| Null model | -581.85 | 1167.70 | 1177.15 |
| Individual variables | -481.86 | 989.72 | 1051.18 |
| Community variables | -517.39 | 1044.78 | 1068.42 |
| Individuals and community variables | **-453.90** | **933.80** | **995.25** |

1. Chakrabarti A, Ghosh JK. AIC, BIC and Recent Advances in Model Selection; 2011.

2. Hawkins DM (2004) The problem of overfitting. Journal of chemical information and computer sciences 44: 1-12.

3. Höge M, Wöhling T, Nowak W (2018) A primer for model selection: The decisive role of model complexity. Water Resources Research 54: 1688-1715.

4. Ding J, Tarokh V, Yang Y (2018) Model selection techniques: An overview. IEEE Signal Processing Magazine 35: 16-34.

5. Myung IJ (2000) The importance of complexity in model selection. Journal of mathematical psychology 44: 190-204.
